# Pain expectations, experiences and coping strategies used by post-operative patients: A descriptive phenomenological study

**DOI:** 10.1371/journal.pone.0298780

**Published:** 2025-06-10

**Authors:** Richard Sakyi, Edward Appiah Boateng, Abigail Kusi-Amponsah Diji, Kenneth Adjei Afful, Vincent Afriyie Nimoh, Philomena Asakeboba Ajanaba, Mabel Dorothy Adjei, Felix Apiribu, Veronica Millicent Dzomeku

**Affiliations:** 1 Nursing and Midwifery Training College, Sunyani, Bono Region, Ghana; 2 Department of Nursing, Kwame Nkrumah University of Science and Technology, Kumasi, Ashanti Region, Ghana; 3 Research Unit.Sunyani Teaching Hospital, Sunyani, Bono Region, Ghana; 4 Department of Nursing, University for Development Studies, Tamale, Northern Region, Ghana.; Queen's University Belfast, UNITED KINGDOM OF GREAT BRITAIN AND NORTHERN IRELAND

## Abstract

**Objectives:**

Post-operative pain(POP) is still an unresolved problem worldwide, including in limited-resource countries such as Ghana. Earlier studies have mainly focused on postoperative pain experiences of patient with little attention to their pain expectations and coping strategies. The current study sought to qualitatively explore pain expectations, pain experiences, and coping strategies used by adult surgical patients to help add patients’ perspectives to surgical pain management.

**Methods:**

A descriptive phenomenological design approach was used to study nine purposively sampled surgical patients receiving care at a regional hospital in Ghana. Participants were individually interviewed before and during the postoperative period to share their opinions on their pain expectations, postoperative pain experiences, and coping strategies. Recruitment and data collection took place between July 8, 2021, and August 30, 2021. The semi-structured individual interviews were audio-recorded, transcribed verbatim, and content analysed to generate themes that described participants’ accounts.

**Results:**

The participants consisted of six females and three males, aged 24–40, who had undergone major surgeries. This study derived three main themes: diverse pain expectations and experiences, post-operative pain effects, and post-operative pain coping strategies. The study revealed that participants had different pain expectations and experiences, and surgical pain affected their activities of daily living and emotions. Participants coped with the postoperative pain by using personal strategies and seeking support from nurses.

**Conclusion:**

Pain expectation of surgical patients affects their post-operative pain experiences. Surgical patients use coping strategies in their post-operative pain management. More needs to be done in reducing surgical patients’ experience of post-operative pain.

## Introduction

Post-operative pain(POP) is a predictable effect of the injury resulting from surgery. It can also be considered an adaptive response that aids healing by limiting movements and other behaviours that can cause additional tissue damage [1,2]. However, post-operative pain can result in several complications, including deep vein thrombosis, pneumonia, delay in healing and discharge and the development of chronic pain [3, 4, 5].

All patients undergoing surgical procedures expect to experience a certain degree of pain; for this reason, post-surgical pain has become one of the most significant concerns for surgical patients [6]. Expected higher post-operative pain by patients pre-operatively has been seen to be associated with the experience of stronger post-surgical pain intensity and severity [7]. Moreover, the experience of post-operative pain is believed to be a worldwide problem [8]. Notwithstanding the improvement in pain management interventions, many surgical patients still experience acute post-operative pain [9]; in developing countries, however, the prevalence of postoperative pain is noted to be high [10]. This brings to bear the need to do more to decrease further the burden of post-operative pain experienced by patients.

Patients’ own pain coping strategies are believed to be one patient-centred approach that has been seen to help minimize the pain intensity in post-major surgery, as when these coping strategies are effective, they help to produce permanent adaptation [11]. Therefore, understanding coping is critical to optimising healthy development and thus providing the needed support that patients may need to manage their post-operative pain better [12].

One of the goals of postoperative pain management is to ensure maximum function and comfort. This can only be achieved when patients become the centre of care by actively participating in all decisions and interventions affecting their care [13]. This is critical in pain management because pain expression is individualised and subjective [14]. Hence, there is a need to look into patients’ coping strategies.

A review of the literature points to the fact that some work has been done on postoperative pain in other areas in Ghana, many of whom centred on nurses and, in some cases, patients:

An explorative qualitative study has been done in a resource-constraint hospital in Ghana to explore how nurses assess and manage POP. The information was gathered using a semi-structured interview guide, and it was found that there was a lack of standard tools for evaluating POP and resource constraints that affected nurses’ management of postoperative pain. [15]. Even though this study is vital in POP management, the study failed to incorporate the expectations and coping strategies of patients in POP this vital because pain expectations are believed to influence pain intensity [7]. Coping strategies can also be used to determine to what extent patients might require other interventions [11].

A study also employed qualitative ethnographic principles through face-to-face interviews to explore patients’ experiences of POP and factors that affect postoperative pain in a tertiary hospital and a regional hospital in Accra, Ghana. The study brought to bear the importance of patient education and the need for healthcare professionals to appreciate the context-specific factors that affect post-operative pain management [16]. Another study in Komfo Anokye Teaching Hospital, Kumasi Ghana, used a qualitative research design with a phenomenological approach to explore patients’ experiences with postoperative pain management. This study brought to bear the fact that nurses play a crucial role in pain management and that as part of pain management, analgesics and non-drug interventions should be encouraged as they will help to reduce postoperative pain with minimal or no adverse effects [17]. These studies, even though qualitatively done, also failed to include the expectations of patients about POP as well as their pain coping strategies.

A study has also employed a descriptive, cross-sectional survey to examine nurses’ knowledge, attitudes and practices regarding post-operative pain management in four district hospitals in Ghana. The study revealed that nurses in these hospitals have inadequate knowledge of post-operative pain management and ineffectively manage postoperative pain, hence the need to increase their theoretical and practical expertise to improve POP management in Ghana [18]. However, This study did not consider patients’ perspectives on pain management since pain is a subjective phenomenon [14]. Looking at pain management from the angle of patients can further help ensure comprehensive and effective pain management, hence the need for the current study.

It is, therefore, clear that regarding pain expectations, experiences, and coping strategies used by patients with post-operative pain, there is a scarcity of information in Ghana and, for that matter, the Bono region. This research, therefore, seeks to explore the post-operative pain expectations, experiences and pain coping strategies used by surgical patients using the qualitative approach. The findings from this research, therefore, will provide clinicians and, for that matter, stakeholders in postoperative pain management further information on the situation of surgical patients’ post-operative pain expectations and their experience of postoperative pain as well as their coping strategies to help add patients’ perspective to surgical pain management.

## Materials and methods

### Study site

The study was conducted in the surgical wards of a regional hospital in Ghana. The hospital has an array of medical services, including outpatient department(OPD), 24-hour emergency care, pediatric and neonatal care, obstetrical and gynaecological care, general medical services, child and reproductive health care, special clinics (eye, ear-nose-throat, dental, mental health), mortuary services, and theatre/surgical care. It has 330 bed capacity and conducted about 1083 major surgeries in 2020. The hospital has four surgical wards (male and female, orthopaedic, and gynaecological). Surgeries which are managed at the male and female medical wards are general surgeries, which include appendectomies, herniorrhaphies, fistulectomies, mastectomies, thyroidectomies and colostomies; surgeries like hysterectomies; salpingectomies, are managed in the gynaecological ward while orthopaedic surgeries are managed at the orthopaedic ward. In total, the staff strength of the surgical wards is one hundred and seven nine (179). They run three shifts, with each shift having about five staff.

### Research design

This study used a qualitative study methodology with a descriptive phenomenological approach. Phenomenology is a form of qualitative research that centres on analysing a person’s lived experiences in the world.

However, there are two varieties of phenomenology: hermeneutic and transcendental [19]. The transcendental (descriptive) used for this research is a pure description of human experiences. The researchers believe they have concrete ideas of the phenomenon being investigated from the onset. Prepositions from the researchers are bracketed, allowing the researchers to explore the phenomena [20] Therefore, the researchers do not seek to interpret the phenomena with their experiences.

### Sampling and sampled participants

The study involved postoperative patients who underwent major surgeries (these are surgeries involving general or spinal anaesthesia). A purposive sampling technique was used to sample nine [9] adult patients from the hospital’s surgical wards. This number arrived after saturation at the seventh (7th) participant, as no new information was coming out. Two [2] additional participants were interviewed to confirm this phenomenon, resulting in nine [9] participants.

Participants were recruited the day before their surgeries. Those who could neither speak English nor Twi were excluded, as were those who could not speak audibly and unconscious patients. Participants’ recruitment and data collection occurred between 8 July 2021 and 30 August 2021.

### Data collection approach

A letter was sent to the hospital’s medical director, who approved it and directed us to its research department. The research coordinator led us to the ward-in charges, who also helped introduce patients in the wards, after which participants were recruited. All participants were given the needed explanations and the purpose of the study, all their questions were answered, and they were informed that they were at liberty to opt out of the survey at any point in time and that their names would not be linked to any information they provided. All participants approached consented to the study.

Before collecting information, written consent was obtained from them. The participant was made to sign or thumbprint the consent form, which an available family member or significant other witnessed.

A semi-structured interview guide was used for data collection and recorded with an Audio Tape recorder. The interview guide included the participants’ demographic characteristics, pain expectations, pain experience, and post-operative pain coping.

Two people facilitated the interviews: RS and PAA. RS conducted the interviews while PAA observed and took notes. RS, the principal investigator, is an experienced nurse and a nurse educator MPhil candidate who has received training in research methods and qualitative data analysis. PAA is also a nurse and an MPhil candidate who has received training in qualitative research methods and data analysis. RS is male, while PAA is female.

The questions were formulated considering the study’s specific objectives. Probing questions were formulated to elicit deep explanations and understanding of concepts.

The interview guide was pre-tested using four participants from the target population. The patients who were used for the pre-testing were all discharged before the main interviews were conducted. This pre-test effectively identified several ways the interview guide could be improved, both through feedback and the experience of actually doing the interview, leading to modification of the interview guide [21].

Most participants were visited the day before the surgery as part of the pre-operative preparation to establish rapport with the patients. The interviews were conducted in two sessions; before the surgery, patients were interviewed on their pain expectations and subsequently within 48–72 hours post-operatively when they had fully recovered from anaesthetic influences and could communicate well. However, in some cases, participants could not be interviewed pre-operatively. In such instances, they were recruited and interviewed after the surgery.

The interviews focused on pain expectations in the pre-operative phase. In contrast, they focused on participants’ pain experiences and coping strategies in the post-operative phase.

The interviews were conducted in English and Twi, in which the participants could express themselves well, and the researchers could also understand them. The interviews were conducted at an agreed time and date and mainly at the bedside of participants, which was conducive for them when there were no interruptions from others. I ensured that RS, PAA, and the participants were the only people present during the interviews. The interviews were conducted as a conversation with probing questions following responses to guide the conversations towards the study’s objectives. The interviews for each participant lasted between fifteen (15) to thirty (30) minutes for both sections. Participants were also provided with enough time when needed for further explanations.

With the full consent of the participants, an audio tape recording of the interviews was made. A unique audio recording application was used on the phone for the recording, after which passwords were put on the phone and application to ensure that the participants’ data were protected from being used only for this purpose and not accessible to anyone. The researchers also wrote down field notes and conducted observations.

### Data analysis

The recorded data were transcribed and analysed using deductive content analysis.

The data collection and analysis were done concurrently until saturation when no new information came out of the interviews. After each interview, the researchers familiarised themselves with the data by transcribing it verbatim after listening to the interview on several occasions, and the transcript was then compared with the audio by listening to the audio and reading through the transcript [22,23]. Two [2] interviews were done in English, while the remaining seven [7] were done in Twi. The interviews done in Twi were translated and transcribed into English by the researchers, after which the transcripts were verified with participants in Twi to ensure that they portrayed precisely what they meant. The data was transcribed using Microsoft and analysed deductively by generating codes, categories, and themes, considering the objectives of the studies. From these themes, the researchers developed a textual description of what the participants had experienced [24].

### Rigour

Qualitative research should meet the following criteria: credibility, transferability, dependability, and confirmability [25].

To ensure credibility, the questions were reframed in different ways during the interview to be sure that the answer truly reflected the respondent’s intentions; member checking was also done, where information obtained was crosschecked from respondents to be sure that the results were accurate picture of the information they gave to the researchers. A field journal was also kept to keep track of all activities during the research process. A thick description of the research context has also been provided to ensure transferability. To ensure dependability, a thick description of the research process was done, and an audit was done where two experienced nursing researchers audited the research process. A reflective journal was kept to ensure confirmability, and two experienced researchers audited the process.

### Positionality of the researchers

This research results from a collaborative effort among registered general nurses in a low-middle-income country with diverse career stages and professional experiences. Some of us are clinicians, whilst others are nurse educators. Our group comprises journal editors, reviewers, faculty, postgraduate students, and postdoctoral fellows. Some of us are nurses who have taken care of patients undergoing surgery, and others have also experienced pain resulting from surgery. All researchers have had training and education in qualitative research and examine the world using the pragmatic philosophical lens, emphasising “what works” under certain circumstances. Because of this, the researchers chose a descriptive phenomenological study design as we wanted to explore the lived experiences of surgical patients about postoperative pain by bracketing our own beliefs and experiences about the phenomenon during the data collection and analysis phases. In doing this, our ultimate goal was to generate new knowledge that helps improve postoperative pain management in resource-constraint settings.

### Ethical consideration

This study was approved by the Committee on Human Research, Publications, and Ethics (CHRPE) of Kwame Nkrumah University of Science and Technology Ghana with reference number CHRPE/AP/259/21. All participants were given the needed explanations, and written consent was obtained from them before collecting information from them. Participants were made to sign or thumbprint the consent form, which was witnessed by available family members/significant others present; all their questions were answered, and they were informed of the fact that they are at liberty to opt out of the study at any point in time and that their names will not be linked to any information they provide.

## Results

### Demographic characteristics of the participants

Nine (9) participants were interviewed for this study, comprising six (6) females and three (3) males. Participants’ ethnic backgrounds included Ewe (2), Gruma (2), Bono (3), Fante (1), and Asante (1). Participants’ ages ranged from 24 to 40 years. The types of surgery that were done included fistulectomy (1), appendectomy (2), salpingectomy (1), hernia repair (1), and hysterectomy (4). Table 1 illustrates this.

**Table 1:** Demographic characteristics of respondents.

Participants	Age	Sex	Ethnicity	Type of surgery
P1	24	M	Ewe	Fistulectomy
P2	34	M	Gruma	Appendectomy
P3	35	F	Bono	Hysterectomy
P4	29	F	Fante	Hernia repair
P5	23	F	Asante	Salpingectomy
P6	39	F	Bono	Total abdominal Hysterectomy
P7	30	F	Bono	Hysterectomy
P8	65	F	Ewe	Hysterectomy
P9	40	M	Gruma	Appendectomy

(Source: field data 2021)

### Organisation of themes

Table 2 below shows the details of the themes and subthemes that emerged from the data. In all, three (3) major themes and eight (8) subthemes emerged from the data.

**Table 2:** Major themes and subthemes.

Themes	Subthemes
**Diverse pain expectations and experiences**	• Expected postoperative pain intensity • Experienced post-operative pain intensity • Sources of information for expected pain • Pain aggravating factors
**Effects of post-operative pain**	• Expected post-operative pain effects on patients • Experienced postoperative pain effect
**Coping strategies**	• Personal coping strategies • Support from nurses

(Source: field data 2021).

#### Diverse expectations and experiences.

This describes the different expectations participants express regarding their pain expectations and experiences. Four (4) subthemes emerged from this major theme: expected postoperative pain intensity, experienced postoperative pain, sources of information for expected pain, and pain aggravating factors. Participants expected and experienced pains of varied intensities.

#### Expected postoperative pain intensity.

Participants were expecting different postoperative pain intensities preoperatively. They expected their post-operative pain to be severe, moderate, or mild. Below are some of what the participants said:

*“I know it will be very painful, yes, so far as there is going to be a cut. Even if you have an injury, you will feel the pain, so equally surgery, you will feel the pain, but the surgery pain will be higher than normal injury” …….* **P1**

*“Oh, I am expecting to feel the pain. I am thinking that after surgery definitely, I will experience some slight pain.”* ……… **P2**

#### Experienced postoperative pain intensity.

Participants also described their post-operative pain intensity, and it was reported that the pain was severe, moderate, and mild. Participants shared their experiences:

*“After the surgery, when the effects of the anaesthesia were wearing off, and I returned to normal, I started feeling severe pain” ……* **P2**

*“The pain was moderate” ………* **P5**

#### Sources of information for expected pain.

Participants’ pain expectations were based on sources which included personal experiences, information received from family members or friends who have gone through similar surgeries, as well as their intuition. Participants shared their experiences:

*“A friend of mine did a similar surgery and it was so painful she was even pregnant and also had the fibroid. She was one of my co-workers. I visited while she was in the hospital, and it was not easy for her, so when I decided to have this surgery too, I discussed it with her, and she narrated to me what she went through. Hmm, so I have all this in mine”…….* ***P3.***

*‘Because I have down some surgeries before and err as I said earlier when you are going in for the procedure, during the procedure, you will not feel anything because of the anaesthesia they will give you but afterwards when the anaesthesia finish working you will start feeling the pain so base on the first experience” ……….* ***P5***.

#### Pain aggravating factors.

Participants also described the factors that aggravated their postoperative pain; for some, lying down increases their pain, while in the case of others, changing position increases their pain; however, in the case of others, getting out of bed suddenly aggravates their pain. Participants shared their experiences:

*...” Right now, if I want to stand up, I must take my time. If I get up instantly fast, then the pain will come” …….* **P4**

*….” Sometimes when you are lying, and you change position too, then you feel the pain more” …….* **P5**

#### Effects of post-operative pain.

This represented participants’ expectations and experiences regarding how the postoperative pain would affect their activities of daily living and how it affected them postoperatively. The study revealed that they actually experienced what they were expecting.

#### Expected postoperative pain effects on participants.

Participants reported on their expected effects of the post-operative pain. Most participants expected the post-operative pain to affect their daily living activities, including bathing, brushing of teeth, walking, and lifting. Participants shared some of their expectations:

“*I cannot do things like bathing and walking as I used to be because of the pain, even I might not be able to brush my teeth without assistance”.* **P7**

#### Experienced pain effects.

Participants reported how they were affected by the post-operative pain. The pain affected how they were able to perform their activities of daily living as most could not bathe themselves unassisted, and others had difficulty getting out of bed and moving about. For some, they could not even sleep because of the pain. Participants shared their experiences:

*… Yes, you know, because of this pain, hmmm, I could not do things like bathing, lifting, sleeping; it was not very easy for me at all. People had even to assist me in bathing and the others….* **P3**

#### Post-operative pain coping strategies.

This theme describes the strategies participants employed in coping with their postoperative pain. Two (2) subthemes emerged for this theme: personal post-operative pain coping strategies and support from nurses.

#### Personal post-operative pain coping strategies.

Participants reported the personal strategies that were used to cope with their postoperative pain. The study reveals that participants use position changes to cope with their postoperative pain. Participants shared their experiences:

*“Yes, I was in pain. I knew that for the pain, I should be able to endure it, so I tried to endure it. I turned myself in bed, so when I turned to the side, the pain reduced small, but when I turned more, the pain returned, then I turned back” …….* **P2**

In addition to the position changes, participants also use other strategies like praying to God, endurance, supporting the site of pain with hand, relaxation, and crying:

*“Hmm, when I am lying down and am trying to change my position, and I feel the pain, I relax, and when I am getting up too, and I feel the pain, I support the area with my hand, so when I was experiencing the pain, then I will lie down and be wailing, and sometimes I will support the area with my hand and was also praying in my head for God to intervene for me to be relieved of the pain.”* **P5.**

#### Support from nurses.

Participants also reported to nurses for assistance when coping with their pain. Nurses offered support by administering pain medications and using words of encouragement to help patients cope with their pain. Participants shared when and how this was done:

*“……. I reported to the nurses, and they gave me another shot injection then, which helped me; yes,* *the nurses also help; they will just come to you and say it is painful, you have to take your time; everything will find they will say nice things to you to calm you down, you to forget your pain”.* ***P4***

## Discussion

The current study sought to explore pain expectations, post-operative pain experiences, and coping strategies used by adult surgical patients. Participants experienced diverse expectations and experiences.

For a better POP management outcome, identifying and managing patients’ preoperative pain expectations is crucial as it has been noted that unrealistic pain expectations can affect patients’ ability and willingness to participate in their care and appreciate their role in pain management strategies [26]. Preoperatively, the study revealed that participants expected severe, moderate and mild pain postoperatively. The findings from the current study are consistent with a study by [27], who posited that some of their participants when questioned pre-operatively, expected to develop moderate to severe acute pain post-operatively. The expectations in the current study were mainly a result of some damaging information about post-operative pain received from clinicians or others as well as their personal experiences, and this can be collaborated by the fact that increased negative pain expectations can be a result of negative pre-operative information and experiences about post-operative pain [26,28]. Preoperative communication should, therefore, provide positive information about pain and its management as this can reduce fear and distress and hence set more positive and realistic expectations [29]. Clinicians and policymakers should therefore, be aware that improving communication between healthcare providers and patients can go a long way in improving patients’ pain management outcomes [30].

Yearly several surgical patients experience postoperative pain with varied intensities [31].

Several factors, including the type of surgery and the pain expectations, are associated with the intensity of post-operative pain experienced [7,26,32]. In this current study, participants went through different types of surgeries and also had varied pain expectations. Participants in the current study experienced pain of varied intensities (moderate, severe and mild pains) post-operatively. The findings from the current study are consistent with other studies [16,26,33], which have also reported that post-operative patients experience pain of different intensities. The agreement of this study with the literature reinforces the fact that the management of postoperative pain continues to be inadequate [8,34]; clinicians and policymakers should, therefore, look at other innovative strategies for managing post-operative pain. The fact that expected postoperative pain has been seen to pose a greater risk to actual post-operative pain experience can also aid clinicians and healthcare practitioners in identifying patients at greater risk for postoperative pain for more intensive post-operative pain management [35].

Findings from this study revealed that the post-operative pain affected how participants were able to perform their activities of daily living as most could not bathe themselves unassisted, others also had difficulty in getting out of bed and moving about, and the post-operative pain also interfered with patients’ sleep. It affected them emotionally which led to some of them crying. This finding is consistent with several studies, which have also concluded that post-operative pains interfere with a patient’s physical activities of daily living including activities in bed (turning, sitting up and repositioning) and activities out of bed like walking, sitting in a chair and standing [34,36,37]. as well as their sleep and emotions [38]. The consistency of current study with the literature can be attributed to the fact that movement restrictions are adaptive responses that patients use to facilitate recuperation and prevent further potential tissue trauma [1,2]. These effects of post-operative pain cannot be underestimated as it has also been reported that immobility can result in the patient developing deep vein thrombosis as well as other complications like pulmonary atelectasis as well as other long-term effects [39,40]. These can delay the patients at the hospital and increase the workload in health facilities with its economic consequences [40].

Policymakers and clinicians should, therefore, see the need for comprehensive pain management interventions to return the patients to his or her normal function and quality [17].

Participants used personal coping strategies and sought support from nurses to cope with their pain. This is consistent with a study by [12], which found that coping strategies can include personal strategy and professional support.

Findings from this study indicate that participants use position changes like turning in bed, stretching, walking and sitting, as well as lying down to cope with their postoperative pain; in addition, they also resorted to supporting the site of pain with their hand. The different position changes participants use point to the fact that people use different strategies in dealing with similar situations. The findings from the current study are consistent with other studies [10,41]. who asserted that patients use position changes to relieve their pain.

Findings from the current study also indicated that participants used other strategies like praying, endurance, relaxation and crying to cope with their pain. Some believed that the pain they were experiencing was normal and something that is usually associated with the surgery; hence, they just had to endure the pain. For some participants’ movements aggravate their pain; hence, they prefer relaxing; the findings from this study are consistent with other studies which have concluded that psychological/spiritual approaches like praying [42],relaxation and social withdrawal [12] are coping strategies used by patients experiencing pain. It has been seen that strategies like expressing emotions and reassuring thoughts are more related to more perceived control and, in turn, better psychological well-being. [43]. These strategies can be attributed to positive beliefs and spiritual support being intended as a basis for hope and hence can justify coping efforts in the most adverse circumstances. [44].

These strategies imply that participants attempted to take control of their pain management. This means that appropriate coping strategies can allow patients to adjust and be ready to tackle the stressor, which may be pain, damage to body tissue or decreased mobility [11]. Clinicians should, therefore, appreciate the fact that specific coping strategies can help augment the management of postoperative pain as this may help in counselling and shaping personalised pain management to help improve patient recovery [45].

Participants also reported to nurses for assistance in coping with their pain; nurses offered support by administering pain medications, engaging them in conversations and activities to divert their minds from the pain, and using words of encouragement to help patients cope with their pain. Nurses are at the forefront of postoperative pain management, as they play a significant role in assessing, implementing, and evaluating pain management interventions [29,46]. Nurses spends 24 hours with the patient; it is therefore not surprising they are the main source of support to participants in coping with their post-operative pain. The fact that patients report their postoperative pain to nurses for support in dealing with their post-operative pain in this study is in line with a survey conducted by [47] in which it was seen that post-operative patients felt nurses were available and hence sought help from them in managing their postoperative pain. Seeking professional support in coping with distress is a healthy coping strategy [12]. Nurses and, for that matter, health professionals should, therefore, be equipped with the needed skills in pain management to provide the needed support to patients in coping with their postoperative pain.

## Conclusion

This study has concluded that surgical patient’s preoperative pain expectations affect their actual pain experience after surgery. Clinicians should ensure surgical patients receive positive information about postoperative pain pre-operatively. Patients who expect to experience severe post-operative pain should be targeted for comprehensive pain management. We have further reaffirmed the fact that post-operative pain continues to be a problem for surgical patients. Surgical patients rely mainly on internal strategies and external support in coping with their post-operative pain. Nurses are surgical patient’s main source of support in dealing with their post-operative pain. Patients’ coping strategies should be incorporated into pain management interventions. Future studies may consider surgical expectations of patients and their impact on their pain outcomes. The effectiveness of pain coping strategies in post-operative pain management can also be explored, as well as nurses’ experiences and perceptions of patient’s pain coping strategies.

### Implication of the study

The findings of this study have implications for nursing education, nursing practice, health policy.

#### Implication for nursing education.

Nursing institutions can base their curriculum on the findings from this study to include an effective pre-operative communication strategy and a robust pain management strategy, taking into consideration patients’ pain coping strategies, to help reduce patients’ pain expectations.

#### Implication for nursing practice.

Based on the findings from this study, clinicians can work towards identifying patients who have high preoperative pain expectations for targeted comprehensive pain management.

#### Implication for health policy.

Policymakers can based on the findings of this study develop an effective pain management strategy including the development of a program for clinical communication to train nurses in effective communication strategies that will equip them with better ways of communicating with pre-surgical patients to reduce or manage their pain expectations.

### Limitations of the study

Due to the study’s method, the findings are limited to this particular setting and cannot be generalized. However, the processes used in the study have been described in detail to make transferability to other settings possible.

## Supporting information

S1 File Consolidated criteria for reporting qualitative studies (COREQ):32-Item checklist. (PDF)

S2 File Participant information leaflet and consent form. (DOCX)

S3 File Interview guide. (DOCX)

S4 File De-identified transcript. (DOCX)
