# Supplementary material for: Pain expectations, experiences and coping strategies used by post-operative patients: A descriptive phenomenological study
Source: PLoS One. 2025 Jun 10;20(6):e0298780. doi: 10.1371/journal.pone.0298780 (PMC12151414; doi:10.1371/journal.pone.0298780)
Supplement: S2 File — (DOCX) [file pone.0298780.s002.docx]

# Participant Information Leaflet And Consent Form

**Title of Research:** Expectations experiences, and coping strategies used by patients with post-operative pain in a regional hospital in Ghana.

**Name(s) and affiliation(s) of researcher(s):** This study is being conducted by:

1. Richard Sakyi- Nursing and Midwifery Training College Sunyani, Bono Region, Ghana.
2. Edward Appiah Boateng: Department of Nursing, Kwame Nkrumah University of Science and Technology, Kumasi, Ashanti Region Ghana.
3. Abigail Kusi-Amponsah Diji: Department of Nursing, Kwame Nkrumah University of Science and Technology, Kumasi.
4. Kenneth Adjei Afful: Nursing and Midwifery Training College Sunyani, Bono Region, Ghana.
5. Vincent Afriyie Nimoh: Research Unit. Sunyani Teaching Hospital,Sunyani Bono Region,Ghana
6. Philomena Asakeboba Ajanaba: Department of Nursing, University for Development Studies, Tamale, Northern Region, Ghana.
7. Mabel Dorothy Adjei: Nursing and Midwifery Training College Sunyani, Bono Region, Ghana
8. Felix Apiribu: Department of Nursing, Kwame Nkrumah University of Science and Technology, Kumasi,Ashanti Region,Ghana.
9. Veronica Millicent Dzomeku:Department of Nursing, Kwame Nkrumah University of Science and Technology, Kumasi,Ashanti Region,Ghana.

**Background (Please explain simply and briefly what the study is about):** Pain is an unpleasant and sometimes unbearable experience which can produce changes in all the systems of the body (Thamilselvam and Pandurangan, 2017). Surgical patients are mostly affected by pain. Since all patients undergoing surgical procedures experience a certain degree of pain, this has become one of the greatest concerns for surgical patients (Málek and Ševčík, 2017). One of the goals of post-operative pain management is to ensure maximum function and comfort. However this can only be achieved when patients become the centre of the care by being actively involved in all decisions and interventions affecting his or her care (Hess *et al.*, 2015). Hence researching into post-operative pain taking into consideration the patient’s, expectations, experiences and coping strategies will go a long way to provide some of the much-needed information to improve the management of post-operative pain.

**Purpose(s) of research:** The purpose of this study is to explore the expectation, experiences and coping strategies used by patients with post-operative pain .

**Procedure of the research, what shall be required of each participant and approximate total number of participants that would be involved in the research:**

The study units will be sampled by the purposive sampling approach. As and when patients are operated upon.The sample size will be dependent on the frequency of the surgical operations performed on patients within the study duration. But the researcher will continue to collect data until no new idea emerges, a phenomenon known as data saturation. Semi-structured interview and audio tape recording will be used to record patients’ responses. Any subject requiring clarification or explanation will be duly assisted.

The surgical ward will be visited on each day of admission of patients for a surgical operation the subsequent day(s), and as part of the admission and pre-operative preparation, the researcher will be part of the detailed rapport establishment. The study will be thoroughly explained to patients and families available. The interview will be conducted in two sessions, prior to the surgery, patients will be interviewed on their pain expectations and subsequently at least 48 to 72 hours post-operatively as this will ensure that patients have fully recovered from anaesthetic influences, and are able to communicate well**.**

**Risk(s):** The study will not pose any risk to you**.**

**Benefit(s):**

It is expected that findings from this study will help improve the care of patients and also contribute to the current literature on pain management. Recommendations from this study will help policy makers in developing good and effective guidelines for post-operative pain management. Stakeholders in nursing education can also utilize findings from this study to help improve the curriculum.

**Confidentiality:** information collected from you will be in the form of audio recording which will be later transcribed, no name will be required from you. Data can therefore not be linked in any way to you. And the transcribed information will be used only for the purpose of this research.

**Voluntariness:** Taking part in this study should be out of your own free will. You are not under obligation to. Research is entirely voluntary.

**Alternatives to participation:** If you choose not to participate, this will not affect your treatment in this hospital/institution in any way.

**Withdrawal from the research:** You may choose to withdraw from the research at anytime without having to explain yourself. You may also choose not to answer any question you find uncomfortable or private.

**Consequence of Withdrawal:** There will be no consequence, loss of benefit or care to you if you choose to withdraw from the study. Please note however, that some of the information that may have been obtained from you without identifiers (name etc), before you chose to withdraw, may have been modified or used in analysis reports and publications. These cannot be removed anymore. We do promise to make good faith effort to comply with your wishes as much as practicable.

**Costs/Compensation:** For your time and the inconvenience, I will compensate you with GH¢10 to show our appreciation for your participation).

**Contacts:** If you have any question concerning this study, please do not hesitate to contact Richard Sakyi on 0546548260.

**Further, if you have any concern about the conduct of this study, your welfare or your rights as a research participant, you may contact:**

**The Office of the Chairman**

**Committee on Human Research and Publication Ethics**

**Kumasi**

**Tel:0322063248 or 0205453785**

# Consent Form

**Statement of person obtaining informed consent:**

I have fully explained this research to ____________________________________ and have given sufficient information about the study, including that on procedures, risks and benefits, to enable the prospective participant make an informed decision to or not to participate.

DATE: ____________________ NAME: _________________________________

**Statement of person giving consent:**

I have read the information on this study/research or have had it translated into a language I understand. I have also talked it over with the interviewer to my satisfaction.

I understand that my participation is voluntary (not compulsory).

I know enough about the purpose, methods, risks and benefits of the research study to decide that I want to take part in it.

I understand that I may freely stop being part of this study at any time without having to explain myself.

I have received a copy of this information leaflet and consent form to keep for myself.

NAME: ______________________________________________________________

DATE: ____________ SIGNATURE/THUMB PRINT: ___________________

**Statement of person witnessing consent (Process for Non-Literate Participants):**

I (Name of Witness) certify that information given to

(Name of Participant), in the local language, is a true reflection of what l have read from the study Participant Information Leaflet, attached.

WITNESS’ SIGNATURE (maintain if participant is non-literate): ____________________

MOTHER’S SIGNATURE (maintain if participant is under 18 years): ________________

MOTHER’S NAME: ______________________________________________________

FATHER’S SIGNATURE (maintain if participant is under 18 years): _________________

FATHER’S NAME: ______________________________________________________
