# Supplementary material for: Pain expectations, experiences and coping strategies used by post-operative patients: A descriptive phenomenological study
Source: PLoS One. 2025 Jun 10;20(6):e0298780. doi: 10.1371/journal.pone.0298780 (PMC12151414; doi:10.1371/journal.pone.0298780)
Supplement: S3 File — (DOCX) [file pone.0298780.s003.docx]

# Semi-structured interview guide

## Topic: Pain Expectations, Experiences, and Coping Strategies used by Patients with Postoperative Pain

## Section A: Demographics

1. Can you tell me about yourself…….

***Probe***: Age, level of education, marital status, religious background, nationality, ethnicity.

## Guiding Questions

2. Tell me if you have ever had any surgical experience

*Probe*: major surgery, minor surgery**.**

3.Tell me why you decided to have the surgery.

***Probe:*** Did you know of any other option aside the surgery?

## Section C: Pain Expectation

# 4. What are your expectations regarding the (intensity, duration, location etc) of the pain that you will be experiencing after surgery?

***Probe***

What informed this expectation that you had? (eg. previous surgery or trauma, etc

What is your expectations regarding how the pain would affect your activities of daily living Bathing, Grooming, Continence, Movements, sleeping, eating, etc.)?

## Section B: Pain Experience

5. Can you please describe the nature of the pain you experience postoperatively?

***Probe***: severity, location, duration, characteristics/ feel (piercing, dull, numb, etc), aggravating factors.

6. Did you experience any other symptoms?

***Probe***: nausea/vomiting, constipation, weakness, itching, confusion, urinary retention.

7. Can you tell me how the pain affected you?

***Probe***: Activities of daily living (Bathing, Grooming, Continence, Movements, sleeping, eating, etc.).

## Section D: Coping Strategies In Post-Operative Pain

8. Tell me the things you did to cope with your pain after the surgery.

***Probe: personal strategies, external support.***

9. How will you describe the effectiveness of your strategies?

***Probe****: Will* you recommend these strategies to someone in a similar situation?
