# Supplementary material for: Pain expectations, experiences and coping strategies used by post-operative patients: A descriptive phenomenological study
Source: PLoS One. 2025 Jun 10;20(6):e0298780. doi: 10.1371/journal.pone.0298780 (PMC12151414; doi:10.1371/journal.pone.0298780)
Supplement: S4 File — (DOCX) [file pone.0298780.s004.docx]

**TOPIC: EXPECTATIONS EXPERIENCES, AND COPING STRATEGIES USED BY PATIENTS WITH POST-OPERATIVE PAIN**

## **Transcript 1**

**Interviewer**: can you tell me something about yourself without your name?

**Interviewee**:

**Interviewer**: Have you ever had any surgery before (minor or major)?

**Interviewee**:I have never experienced it before, yes for minor yee, my stomach, at that time I was having severe stomach problem, and at that time they said there is something they have some traditional name for it they said koraa or something like that so they sent me to a traditionalist and they used normal blade to make some incisions around my abdomen. Apart from that I have not experience any other thing.

**Interviewer**: Why did you come for this surgery?

**Interviewee: yes** since I had this problem I decided to do it so that I will be free.

**Interviewer**: Did you know any other solution to your problem aside this?

**Interviewee**: oh no I have being given a lot of flucloxacillin several times I went to a pharmacy shop to complain of this problem for the fluclox infact I took it for a longtime but it was not resolving so I decided to come to the hospital which they diagnose as fistulectomy so I know surgery is the only way out now.

**Interviewer**: So what is your pain expectation with regards to the surgery?

**Interviewee:** I know it will be very painful so I think one week to two weeks it will be ok,yes so far as there is going to be a cut even you yourself if you have an injury you will feel the pain so equally surgery too you will feel the pain but the surgery pain will be higher than normal cut so I don’t have any problem but I know because I want to be free I don’t have any problem with the pain.

**Interviewer**: so what is the source of this expectation?

**Interviewee** No I know so far I as am coming to do surgery It will be painfull,as even for this cut it was painful,

**Interviewer**: what are your expectation on how the pain will affect your activities of daily living?

**Interviewee** Yes I know after the surgery I can’t be moving up and down as expected

**Interviewer**: Were you expecting the pain to be so severe?

**Interviewee** I was expecting that I will feel the pain but just that I have not experience the pain before but right now I have experience it so next time when am talking about the pain I will know what to say.

**Interviewer**: can you tell me your post-operative pain experience?

**Interviewee:ohhh** Is only this morning that I started feeling the pain because yesterday they gave me an injection so after the medication worn off the I started feeling the pain., it was severe ,around 3 :30 -4 am I was even crying but after the injection I was ok.

**Interviewer**: So where were you feeling the pain

**Interviewee** Where they did the surgery and at my back the spinal where they injected.

**Interviewer**: Did you do anything that made the pain worse.

**Interviewee:** Nooo

Interviewer:can you tell me if you experienced other symptoms aside the pain?

**Interviewee**:oh no for me it was only the pain, did not experience any other problem.

**Interviewer**: Can you tell me how the pain affected you as in your activities of daily living?

**Interviewee** Hmm now I can’t bath, and roam about as I use to because of the pain and if not because of the pain,I will I will be somewhere by now.

**Interviewer**: so what did you do to cope with the pain

**Interviewee** When it started I was lying down flat like this and I was feeling the pain will be there but the moment I turn myself like this then I will be Ok sometimes when am lying flat like this then is ok but the moment I turn myself like this then I fell the pain,so when I position myself in certain way and I feel am ok then I want to remain like that so I don’t want to turn so that I wil feel the pain

**Interviewer**: Aside the turning did you do any other thing,

**Interviewee** That was the injection,so by that time I was not having the injection so I called the nurse and I complain to her and they gave me the injection.

**Interviewer**: So how effective was your strategies

**Interviewee** I think theirs were effective cos I was turning all over but the moment I had the injection I was very ok.

**Interviewer**: So when somebody is in this situation will you recommend what you did to him or her?

**Interviewee** Ohh I will recommend the injection for the person than turning yourself my situation MY turning myself I could see that am ok but your situation maybe you can see that you are worsening the case ,so I will recommend the injection .

Thank you very much

**Interviewer**: But if you compare what you were expecting to what you experience which one is higher

**Interviewee** I Think my expectation was higher .it didt go into that range I was expecting.

## **Transcript 2:**

**Interviewer**: Can you tell me something about yourself without your name?

**Interviewee:**

**Interviewer**: **Have you ever had any surgery before (major or minor)?**

**Interviewee:** Yes on my leg we were playing football and I had a cut so they have to suture it in Kumasi.

**Interviewer**: **So why did you decide to come for this surgery**

**Interviewee:** I was in K I was working there so one morning I worked up and my abdomen was really paining me and I collapse, by the time I became aware I was at the hospital and upon investigations I was told I had appendix so doctor made us to take a scan at T JEE and that one also proved that it was appendix and looking around there was no family member in K but for the Doctor he transferred us to go to a certain hospital so they can do the surgery because I had no family member around to assist me I requested to be referred to T where I have my relatives and the Doctor agreed and I was referred to T so open reaching T we decided to come to S.

**Interviewer**: **Did you know of other treatment options aside this surgery?**

**Interviewee:** When they said it for me I asked them if there is no drug to treat it and they said they can give medications but it might not help so the surgery is what will help to make everything ok.

**Interviewer**: **What about when you came home too?**

**Interviewee:** We did not even go to the house straight from K we came here.

**Interviewer**: **So what was your pain expectation towards this surgery?**

**Interviewee:** Oh I was expecting to feel pain I was thinking that after surgery definitely I will experience some slight pain but then it will be ok.

**Interviewer**: Where exactly where you expecting the pain?

**Interviewee:** Oh below by umbilical area that was where I was expecting to feel the pain.

**Interviewer**: What was your expectation with regards to the duration of the pain?

**Interviewee:** I was not expecting it to last long but for how long I don’t know.

**Interviewer**: **What is the source of your expectation?**

**Interviewee:** For me because there is going to be a cut definitely I knew that there is going to be pain,

**Interviewer**: **What were you expecting regarding post-operative pain restrictions on your activity of daily living?**

**Interviewee:** Oh no I was not expecting anything like that.

**Interviewer**:  **So after the surgery what was the pain experience?**

**Interviewee:** After the surgery when the effects of the anesthesia was wearing off and I return to normal I stated feeling severe pain that very day I could not sleep here.

**Interviewer**: **how long did the pain last?**

**Interviewee:** Oh it was that very day but the next day it was ok but when I get out then I feel the pain at the site of incision and also my other side and my waist

**Interviewer**: **Did you experience others symptoms aside the pain?**

**Interviewee:** Oh no I did not experience anything.

**Interviewer**: **What did you do to cope with your pain?**

**Interviewee:** Yes I was in pain I knew that for pain I should be able to endure it so I tried to endure it, I turn myself in bed, so when I turn to a side the pain reduces small but when I turn more the pain returns then I will turn back

**Interviewer**: Did you do any other thing that helped you?

**Interviewee:** Aside the turning I did not do any other thing

**Interviewer**: **Did others also do something to help you cope?**

**Interviewee:** Yes I had some drugs so they told me when am in pain they will give it to me so when I was in severe pain they gave me the injection and I became ok.And the nurses was also always present at my bedside so that gave me the confidence that everything will be ok.

**Interviewer**: Was your coping strategies helpful to you in managing the pain?

**Interviewee:** Oh yes it was helpful.

**Interviewer**: **Will you recommend these strategies to another person in a similar situation?**

**Interviewee:** Oh yes and I will encourage them that I know the pain I went through but as far as God is alive it is not anything so serious and the nurses too have patients for people so I will encouge the person to do it and I can say something around what I did that helped me too.

## **Transcript 3**

**Interviewer: Can you tell me something about yourself without your name?**

**Interviewee:**

**Interviewer: Have you ever had any surgery before (minor or major)?**

**Interviewee:** Oh yes my leg I had a cut there and so was sent to hospital for and they sutured the place.

**Interviewer: Why did you come for this surgery?**

**Interviewee: oh I had a lower abdominal problem and so I visited the hospital** I was told I had fibroid and it has enlarged so I needed the surgery.

**Interviewer: How did you get to know it has grown big?**

**Interviewee** Oh I was told after I visited hospital the doctor made me take some scan and the report indicated that it has enlarged and that it was even multiple and so the doctor said surgery needs to be done.

**Interviewer** Did you know of any other treatment alternative aside the surgery?

**Interviewee:** oh yes some people said traditional medicine can be helpful and so they encouraged me to try but for me I was not interested in that I prefer the surgery to those things.

**Interviewer: can you tell what you were expecting with regards to pain(intensity,duration,location)?**

**Interviewee** Oh I yes I expected it to be very painful, you know the place where they will cut no I know that is where the pain will be.

**Interviewer:**  Can you tell me the source of this your expectation?

**Interviewee:** A friend of my did a similar surgery and it was so painful she was even pregnant and also had the fibroid. She was one of my co-worker I visited her while she was in the hospital and it was not easy for her ,so when I decide to have this surgery too I discussed it with her and she narrated to me what she went through hmm so I have all this in mine.

**Interviewer: Can you describe your post-operative pain experience (intensity,duration,location)?**

**Interviewee:** For the pain it was very painful. When the anesthesia worn off it was not very easy sleeping self was difficult infact the place where the incision was made was paining me a lot.

**Interviewer: How did the pain affect your activities of daily living?**

**Interviewee** Yes you know because of this pain hmmm I could not do things like bathing, lifting, sleeping,it was not very easy for me at all people have to even assist me in bathing and the others.

**Interviewer: can you tell me what you did to cope with your post-operative pain**?

**Interviewee:** Oh for me when I walk around the pain subsides and when I lie down the pain increases so I usually walk around.We were encouraged to get out of bed early so I try to get up and walk for me when am walking the pain is OK.but immediately I lie down the pain increases to I prefer walking.

**Interviewer:** Can you tell me what others also did to help you cope with the pain?

**Interviewee** O yes they gave me medications and counselling was also done.So when I was in Pain the nurses gave me some medications after which it was ok they also tell you encouraging words to take you mind off the pain.

**Interviewer: can you tell me more about who came to counsel you and how it help you cope with the pain?**

**Interviewee** Oh psychologist and my family member. The psychologist came here before we went for the surgery to Counsel us in fact I was a little afraid of the surgery, so after the counselling, I was relieved and better prepared for the surgery. So it was not about the pain perse,but it helped me in my fear.

**Interviewer:** Will you recommend your interventions to another person coming for a similar surgery?

**Interviewee**: oh yes once they were helpful to me I think is I will also recommend same to the another person as it might also help her.

## **Transcript 4**

**Interviewer:** Can you tell me about yourself without your name?

**Interviewer:** So tell me why you decided to have the surgery.

**Interviewee** Ahhh this is not the first time of doing this kind of surgery I had the first one at the holy family hospital B but the second one the pain was unbearable yea the pain was unbearable I took it there back and the information they gave me I relax about the information they gave me so latter on when I relax the pain was unbearable then my husband decided we should bring it here and find out what really the problem is so when we came they told us that is an incisional hernia so they have to do it as early as possible so the pain because the lining of the stomach os torn and the intestines is out and the pain that am feeling that is unbearable the blood could not flow well where the intestines are out so I have to do the surgery because the more am feeling the pain then that place will eventually die and it will cause another problem so I asked them to do the surgery.

**Interviewer: Did you know of any other treatment option aside the surgery?**

**Interviewee:** Oh for me no

**Interviewer: So, from what you are saying, you have ever had surgery before?**

**Interviewee:** Yes, the same thing as my first born. They said it is called epigastric hernia hahaha so when I came here then, they told me it was an incisional hernia.

**Interviewer: so what is your pain expectations towards this surgery?**

**Interviewee** Hmmm initially I was not thinking about the pain but I was scared what my mind was going through was not actually the pain but I know I will go through pain and because it was an operated thing I know it will be painful it will be severe but not as much as.

**Interviewer: can you tell me the source of your expectation?**

**Interviewer:** Hmmm Nobody told me about the pain but I was errm I actually comparing the pain from the first one to this one is a little bit painful than the first one.

**Interviewer: can you tell me your expectations regarding how the pain will affect your activities of daily living?**

**Interviewee** :That’s what I was saying this is not the first time am doing the surgery so I know if I do it I will not be able to bath, I just clean myself, not to the extent of pouring water on the body and taking your bath I know I can’t do that, for the brushing I can brush.

**Interviewer: Please can you describe the nature of the pain you experience posnt-operatively(severity,location,duration)?**

**Interviewee**:Comparing it to birth this is more painful than birth yes delivery,am feeling it around the whole stomach is an incisional hernia on top of the navel is on top of the navel where the incision was made, comparing the first day and today is find, today is find because today am able to stand up. Walk…go do a whole lot it is far decreasing

**Interviewer: what we the pain aggravating factors?**

**Interviewee** Hmm I think yesterday after the surgery I threw out yes I vomited and it was painful, right now if I want to stand up I have to take my time if I d it instantly fast then the pain will come

**Interviewer: Are there things that reduces the pain**

**Interviewee** Hmmm Unless I lie down then I will turn side by side, I lie here then I will turn and go to the other side.

**Interviewer: Any symptoms aside pain,**

**Interviewee** Yes I vomited and they said it’s the anesthesia aside that I have not experience any other thing.

**Interviewer: How did the pain affect you**

**Interviewee** Right now concerning daily activities I can’t really tell you because rignt now am still lying not going up and down when am going to the wash room they have to help me and I also clean myself in bed .

**Interviewer: can you tell me what you did to cope with your pain after the surgery?**

**Interviewee** :Just sleeping off just lying down then I just hamming myself then I just dose off when the pain comes I don’t want to talk I just lie down down then I endure it.And if am tired of lying I will just sit.I also report to the nurses so for example yesterday when the pain came I reported to the nurses and they gave me another shot injection then it help me, yes the nurses also help they will just come to you is painful you have to take your time everything will be find they will just say nice things to you to calm you down you to forget your pain.

**Interviewer: Did these interventions help you in coping with your pain?**

**Interviewee**: oh yes they really helped to reduce my pain.

**Interviewer: Will you recommend your strategies to others in a similar situation?**

**Interviewee** :Oh ye I will I will just tell them how surgery is and if you want to calm down your pain you just be qiet,bring out your favorite songs and if you cant sing it out maby you memorizes or you sing it inside you,so the best thing for you is to be alone at that moment.

## **Transcript 5**

**Interviewer: Please can you tell me something about yourself without your name?**

**Interviewee**:

**Interviewer: Have you ever had any surgery before?**

**Interviewee**: Oh yes it was a lipoma so they did the surgery at koforidua regional hospital.

**Interviewer: Why did you come for this surgery?**

**Interviewee**: I didt know anything about it I was feeling my natural menses so I was feeling pain and I came to the hospital and they requesting for lab so it was the lab that shown that I was pregnant and it was in the fallopian tubes either I go medical or surgery so they gave me two options so they requested for labs I did bet a eck kidney function tests and liver function test but based on the beta ecg it was very high if am to go medical then it will take long and it can rapture so I choose surgery.

**Interviewer: Did you have any other option.**

**Interviewee**: No.

**Interviewer:** What was your pain expectations (intensity, location?)

**Interviewee**: Actually I was panicking I was afraid although I have being at the theart before but that one it was just a minor thing but going into an invasive place I was very afraid I even cried so it was the nurses who consoled me that though it was a major surgery but I it is not anything everything will be find there was a little fear though. Oh know after surgery you will feel pain but during the procedure because of the anesthesia they give you will not feel pain but I was expecting a medium pain.

**Interviewer: Can you tell me the duration of pain?**

**Interviewee**: Maybe A day after and site of injure actually I know the place they are going to do the procedure that is where you will feel the pain

**Interviewer: Can you tell me the sources of your pain expectations**

**Interviewee**: Because I have down some surgery before an err as I said earlier when you are going in for the procedure during the procedure you will not feel anything because of the anesthesia they will give you but afterwards when the anesthesia finish working you will start feeling the pain so base on the first experience.

**Interviewer: What were your expectations regarding how the pain is going to affect you?**

**Interviewee**: Err like bathing, lifting of heavy loads and the rest I think I will have difficulty in doing those things.

**Interviewer: Can you tell me about your post-operative pain experience? Intensity location**

**Interviewee**: Ohhh it was not easy it something that is there no matter what it wasn’t easy but after sometime I recovered the pain was moderate and it was located at where they did the surgery ,this one I don’t know it you have headache before it was like something that is breathing.

**Interviewer: can you tell me the aggravating factors of your pain?**

**Interviewee**: Hmmm the next day I was asked to wake up and I was finding it difficult but by supporting by hand there I was able to walk. Sometimes when you are lying and you change position too then you feel the pain more.

**Interviewer: So did you have any other symptoms aside the pain**

**Interviewee**: Yeerrrr it was like my stomach was bloating when I complained to doctors they said when I start fluctuating it will be ok. So they asked me to do a little exercise like walking, standing and after doing after doing that that It was ok, during the surgery I vomited

**Interviewer: Can you tell me how the pain affected you?**

**Interviewee**: Well it has not affected me as such but me being able to bath previously have being limited and the way I used to walked before the surgery too now I have to walk slow, for sleeping I can sleep but just the first day the because of the pain I couldt sleep,

**Interviewer: can you tell me what you did to cope with your pain(personal and external support)?**

**Interviewee**: Hmm when am lying down and am trying to change my position and I feel pain I relax, and when am getting up too and I feel the pain I support the area with my hand,so when I was experiencing the pain then I will lye down and be wailing,and sometimes I will support the area with my hand and was also praying in my heard for God to intervene for me to release the pain.

**Interviewer: did you get any external support to cope with your pain?**

**Interviewee**: Err Ok that very day it was late so immediately I was brought from the theatre the nurses left they all left but the nurse day morning the other nurse, one of the nurses asked me to try and get out of bed so that they will know that after the surgery am recovering after wards they did bed bathing for me and the doctors also came to examine me.sometime the nurses will also give you a medication to stop the pain. And then they will also be charting with you so that your mind will not be on the pain and some will come and encourage you others too, some of the nurses has gone through the same surgery so they will use their experience to encourage you

**Interviewer: So how effective was your coping strategies.**

**Yeee If somebody is going through the same procedure will your recommend. Same to them.**

**Interviewee**: Ohh that was I did to help me it help me and equally the nurses also told me that when am trying to get up and I feel pain I should support the place with my hand and try push it in and latter relax small thtand it helped me a when I did that.

## **Transcript 6**

**Interviewer: Can you tell me something about yourself without your name?**

**Interviewee**:

**Interviewer:** Have you ever had any surgical experience before (minor or major)?

**Interviewee**: No I have never had any surgery before.

**Interviewer:** **What made you decide to have this surgery?**

**Interviewee**: When I see how my body is doing me is not like previously ahha if you consider the slight pain you are going through and you don’t take time it will persist and what the doctors will say too you obey so they can help for everything to be Ok

**Interviewer:** **So what did they say is wrong with you?**

**Interviewee**: They said I have a problem in my womb so unless they remove it so in the beginning the doctor asked me how many children I have and I said four so when I mention my age he said it will be better they remove everything if not it can reoccur.

**Interviewer:** **Did you know of any other alternative treatment?**

**Interviewee**: Oh no in the beginning I went to a private hospital I taught it was malaria so when I went and the Doctor did some blood test and later said I should go and do urine test because what he is looking for is not in the blood test so after the urine test he then said it was pregnancy so he said two weeks I should then go for a scan and bring it so when I went for the scan they said nothing is there lie that so he again gave me two weeks and directed me to a place where I should go for the scan so after that scan too the doctor said I have an infection in the womb so he referred me to regional hospital.

**Interviewer:** **Can you tell me your pain expectation?(intensity, location etc)**

**Interviewee**: I have not done some before but I know you will not take easy like that because when you even get a cut it is so painful, as for the intensity unless you are done with the surgery so I knew it will be painful but did not know exactly how it will be like.

**Interviewer:** **What were your expectations on the effects of post-operative pain on your activities of daily living?**

**Interviewee**: For me it was like some of my friends have already done some so they told me when they are done with the surgery and the effects of the anesthesia is gone it will be painful so you yourself you have to contain the pain so if you say because of the pain you will not get up then it will take long before you will be ok.So I will take it like when you are in labour,when it get to that stage nobody can do something to help you unless you are done with the delivery before you will be ok so that was how I took it.So for me I did not consider that there is something I could not do.

**Interviewer:** **Can you tell me something about your pain after the surgery?intensity,location ,aggravating factors?**

**Interviewee**: When I return from the surgery when the effects of anesthesia was wearing off, I felt very severe pain and because of the pain my whole body was not ok,it was really painful so I even asked myself so if it is like this then tomorrow how will I get out of bed,for the site of pain it was within the upper and lower abdomen that was where I really feel the pains,for the legs the effects of the anaethesia had not completely worn off so it was less painful, but the site where the incision was done I felt the pain .for the duration it lasted small.

**Interviewer:** **Did you experience any other symptoms aside the pain?**

**Interviewee**: When it happen like that my temperature was high and when the checked the Blood pressure to it was high so they even asked me whether I had hypertension, but yesterday for sleeping if I say I was able to sleep then am lying it was because of the pain and how I wanted to sleep too I could not get it that way so my lower abdomen was also paining me,so it was the evening that I was able to sleep.

**Interviewer:** **Can you tell me how the pain affected you?**

**Interviewee**: For me I did not see it that way because I was in pain, as I was lying there like that I was in tired so I wanted them to turn me for air to pass my back but when I said it the nurse said if I can do it myself then I should do it but because of the anaesthesia my legs too I could not lift them well so when I raise myself small then I will put my hand at my back so air can pass though my back small.

**Interviewer:** Can you tell me what you did to cope with the pain after the surgery?

**Interviewee**: Hmm you can’t do anything about it when it got to sometime you have not plan to cry but you will see yourself crying ,but I was also turning myself small when I turn left then I will turn left.I was also murmured

**Interviewer:** **What did others also do to help you cope with your pain?**

**Interviewee**: When everybody comes then he will be giving you words of encouragement that I should be patient after sometime the pain will go away I was also thirsty so I wanted to drink water when I told the nurse she said she will attend to me soon then later on when she pass by again and I asked her again then she will so oh she is coming, I relished she was doing so to take my mind off the water because if she says she will not give it to you she don’t know what you will do.They also sometime will come and give me some medicine intravenously and it helped to reduce the pain

**Interviewer:** **Can you tell me how effective these interventions were?**

**Interviewee**: Ohh what everybody did was helpful. Because the person he is the one who is always doing the work so he knows what can help you, because sometimes you may prefer something but if they give it to you it might not help so sometimes they will encourage you small.

**Interviewer:** **So will you recommend what you did to somebody in a similar situation?**

**Interviewee**: Ohh when the person asked you should tell her is nothing just that you have to go through small pain so because the person have not done some before he might not see what is in it but if you get here and afterwards then you will feel what is inside you can get up and say they have you are going you see even in the house your mother will not tell you delivery is painful but when the day comes you will see it yourself. This pain is small severe than even delivery because with delivery you go through it once and that will be all but this one you will even after three days the pain can still be there even up to one week.

## **Transcript 7**

**Interviewer: Please** can you tell me something about yourself without your name?

**Interviewee**:

**Interviewer**: Why did you come for this surgery?

**Interviewee:** oh I was having irregular menses and I taught I was pregnant, but after doing a pregnancy test I realized that it was not pregnancy. and tried many options that friends and others recommended but all was not working so decided to come to the hospital for check-up and upon several investigations I was told I had fibroid and hence will require surgery I it was growing bigger and bigger, so upon the doctor’s recommendation, err I decided to come for the surgery so that I could be free.

**Interviewer:** please did you know of any other treatment option aside this surgery?

**Interviewee:** No I did not know of any since, in the initial stages I tried many options which were suggested to me by friends and in some cases family members but they all did not work so for now I am not interested in pursuing that course again am certain on the surgery as the best option.

**Interviewer**: Have you ever undergone any surgical intervention before,(major or minor).

**Interviewee**: oh noo I have not undergone anything as such.

**Interviewer:** Were you expecting to experience pain after the surgery?

**Interviewee**: Oh yes even when you get a knife cut it is painful. so for me I know am going to experience pain even you see when you are cooking and you have a knife cut do you see how painful it is so comparing that once with a surgery I know that will also be painful.

**Interviewer:** can you tell me how you were expecting the pain to affect your activities of daily living?

**Interviewee**: Yes I know after the surgery I cannot do things like bathing and walking as I use to be because of the pain, even I might not be able to brush my teeth without assistance. But after sometime this will go away and I will be able to resume my normal activities gradually.

**Interviewer:** After the surgery can you tell me about the nature of the pain you experienced (severity, duration, location)

**Interviewee**: Infact for the pain it was very painful, the pain was on and off, and it was located around where the incision was made radiating to my back.

**Interviewer:** can you tell me what other symptoms you experienced aside the pain?

**Interviewee**: oh for me I did not have any other thing like that all that I was concerned about was my pain and I did not see or fell anything like that, that is for me infact I dint know of others, people say sometimes they vomit and others but for me I did not experienced anything like that.

**Interviewer: can you tell me how the pain affected you (activities of daily living? Interviewee**: mmm,eii because of the pain when I want to stand it was difficult, I could not do many things oo even I could not bath myself they have to come and clean us Standing, could not bath.

**Interviewer:** Now can you tell me what you did to cope with the pain you experienced after the surgery?

**Interviewee**: Yes when I turn myself to one side then the pain reduces small.so infact that was what I was doing when I was in the pain.

**Interviewer:** Aside the turning did you do any other thing to cope with your pain?

**Interviewee**: No infact because of the pain they told me I could not be taking more pain medications hence I understood that I have manage with the pain myself

**Interviewer:** can you tell me which people told you that?

**Interview:** Oh it was the nurses, yes they were very helpful to me with their words and their actions so with their words of encouragement I had to manage and endure the pain?

**Interviewer:** Will you recommend these strategies you used in coping with your pain to others in a similar situation?

**Interviewee**: Everybody is different hence people have different ways of managing their pain, for me I will advise that the surgery is painful so when they go for the surgery they will see what it is

**Thank you very much.**

## **Transcript 8:**

**Interviewer:** Please can you tell me something about yourself without your name?

**Interviewee**

**Interviewer: Can you tell me why you came for this surgery?**

**Interviewee**: What happen was that my second born when he got two years we were at D N that was where my husband was working so I went there and stay there for about six months so he was transferred to Sunyani when he was two years old I saw that something have emerge below me like an egg I did not know what it was, so after two years in Sunyani my husband was again transferred to D so there my husband told me to bring it to hospital so I came to Sunyani,regional hospital, we had stayed in sunyani here so when I came I met Doctor D he made me buy a drug and he used it to push the thing back so after sometime my menstrual period became own and of, and latter stopped so even my husband became angry that it was as a result of the hospital that my menstruation has stopped because I was not done with delivering my children so he was worried so he made me write a letter to doctor F for him to know what exactly he did for my menstrual flow to stop but he also said the people are many so he cannot recollect, so I was there ahh se I told you my child will have twenty years this year so I was there when I lie down the it retracts but when I get up then it prolapse so this Easter mother I went to my child in Kumasi after which I saw that the thing has prolapse in-between my thighs and I could not do anything and I was experiencing general bodily discomfort so my husband told me to bring it here so when I came even the tip of the uterus was swaws so they wrote a drug for me and after taking it he wound healed so Dr A scheduled for 9th June to come for the surgery but it was disturbing me so I wanted them to remove it so I will be free so to came here on Tuesday and they gave me bed

**Interviewer:** Did you know of anything that could have be done aside the surgery?

**Interviewee**: Okkk you see when it happen that way they said if I had come earlier, it is about eighteen years oo so it has being with me all this while ooo so if I had come earlier, when I sleep you will not see it but when I walk then it prolapse.so when I came the Doctor said they can remove it for me if am done with delivery but for me the last bone if even 18 years. So I was done with delivery so I wanted them to do it for me.

**Interviewer: What is your pain expectation towards the surgery (severity, location and duration?**

**Interviewee**: You see when they touch you with a knife definitely you will feel pain , you see those of us who have given birth we understand, I gave birth some time ago and episiotomy was done so I experience pain small before it went down so so for pain once they touch you with a knife you will fell it.

**Interviewer: So what is the source of your expectation?**

**Interviewee**: Oh yes by all means it will come, but sometimes we brush it off.so when it happens like that if someone it having a similar problem you can advise her. And even for me one of my sisters have ever done some before and I went and took care of her that one it was fibroid, it was not easy too so I was the one doing the up and dones and as for Accra you have to take about two cars before you reach 37 and the rest but even though the husband was there because she was my senior sister I have to do all the things so I could go for drugs.

**Interviewer: What is your pain post-operative experience?**

**Interviewee**: As I said I went and stayed with my sister in Accra and they did a surgery that one was fibroid I did not enter the theatre so I did not know how the place was so when they sent me to the theatre many machines were there so they made me aware that because of the pain they will inject me at the back so that I that I will not fell the pain so I saw that they have killed all my too lower limbs so I could not lift my legs so they were asking me questions ahhhhh then I went off and did not see anything again so when they were bringing me to the ward self I did not see anything until I regain consciousness ,I did not feel much pain though but intermittently I will fell some slight pain in my lower abdomen so this one too they did it inside so they did not cut me so intermittently I will feel lower abdomen pain then they will give me an injection for the pain to reduce. And sometimes too they insert a suppository and then I will be Ok.

**Interviewer: Did you experience any other symptom aside the pain?**

**Interviewee**: Yes yesterday I loss appetite but today its ok, when I walk small it was Asif am tired.

**Interviewer: How did the pain affect you with regards to your activities of daily living?**

**Interviewee**: Oh for sleeping I was able to sleep but in the first day the nurses came to clean as up but from there am able to walk and to the bath room, fetch water I can clean myself too and do other things.

**Interviewer: So was there something you wanted to do but then the pain prevented you?**

**Interviewee**: Oh no there was nothing like that everybody and his or her problem but for me it was ok all that am waiting is for the wound to heal. I was able to do whatever I wanted to do.

**Interviewer:** So when it was paining what did you do to cope with the pain?

**Interviewee**: Oh I will lie down and when the nurses are coming then I will also inform them that am having some discomfort there so they will then come and insert the suppository and I will be ok

**Interviewer: For some people when they are going through pain,they do something in order to cope with the pain did you do anything like that.?**

**Interviewee**: Yes that is what I was saying I will lie down small so when they come then I will call them I know it’s because of the wound that is why the pain is there but when the wound heals I sure I will be ok,and I also turn myself small. And for the prayer too we pray.

**Interviewer:** What you did to cope with the pain will you encourage someone else to do same?

**Interviewee**: Oh yes I will encourage them do same

Thank you very much

## **Interview 9**

**Interviewer: Can you tell me something about yourself without your name?**

**Interviewee:**

**Interviewer:** Have you ever had any surgical experience (minor or major).

**Interviewee:** Oh no I have not.

**Interviewer: Why did you come for the surgery?**

**Interviewee:** mm the thing appeared here and it will swell up this place it was a hernia so it will come swell there and after some time it will go off.

**Interviewer:** Did you know of any other treatment option?

**Interviewee:** No when they did this one and it came again, something was here, so the hernia was done and they said something else was there and it was piecing my intestines mmm so when they finished this after a month and week when I come for dressing then I informed them that my here when food gets into my stomach then is painful so when I went for the scan I also did not have money so I was transfer here, so when I came here they said there is something here they have to perform the surgery if not it will worry me.

**Interviewer: What was your pain expectation? (Severity, duration, location)**

**Interviewee** Yess I was expecting the pain to be severe. Where the incision was going to be made is where I know the pain will mostly be and for how long I don’t know exactly but I know it will not keep long and I will be ok

**Interviewer:** What is the source of this expectation?

**Interviewee:** Because of the knife they will use to cut me.

**Interviewer: can you tell me the nature of the pain you experience after the surgery(severity,location,duration).**

**Interviewee:** It was very painful for about two days,the site where the incision was done ,it was burning

**Interviewer:** How did the pain affect you with respect to your activities of daily living?

**Interviewee:for that one**  Oh I could not go to toilet,and the abdomen was tense I could not pass flatus too.I could not also get up because of the pain also I could not bath on my own.

**Interviewer: can you tell me how you were able to cope with the pain after the surgery?**

**Interviewee: when the anesthesia worn off and I was in pain** I was stretching myself and turning myself in bed. Infact this was mainly what I was doing and it helped me cope with the pain somehow

**Interviewer: can you tell me what others also did to help you cope with the pain?**

**Interviewee:** The nurse also inserted something in my anus and it help the pain to subside

**Interviewer:** Will you recommend your strategies to others in a similar situation too?.

**Interviewee:** Yes I will encourage them to be patient it will go when I did my that was how it was. When they do this maybe it will pain you but by the grace of God it will go.

Sometimes where you are turning it will be difficult and when you want to get up too it will be difficult.
